# Retrograde Labeling Illuminates Distinct Topographical Organization of D1 and D2 Receptor-Positive Pyramidal Neurons in the Prefrontal Cortex of Mice

**DOI:** 10.1523/ENEURO.0194-20.2020

**Published:** 2020-10-22

**Authors:** Sara M. Green, Sanya Nathani, Joseph Zimmerman, David Fireman, Nikhil M. Urs

**Affiliations:** Department of Pharmacology and Therapeutics, College of Medicine, University of Florida, Gainesville FL 32608

**Keywords:** corticostriatal, dopamine receptor, motivation, prefrontal cortex, psychiatric disorder, topographical

## Abstract

The cortex plays an important role in regulating motivation and cognition, and does so by regulating multiple subcortical brain circuits. Glutamatergic pyramidal neurons in the prefrontal cortex (PFC) are topographically organized in different subregions such as the prelimbic, infralimbic (IL), and orbitofrontal and project to topographically-organized subcortical target regions. Dopamine D1 and D2 receptors are expressed on glutamatergic pyramidal neurons in the PFC. However, it is unclear whether D1 and D2 receptor-expressing pyramidal neurons in the PFC are also topographically organized. We used a retrograde adeno-associated virus (AAVRG)-based approach to illuminate the topographical organization of D1 and D2 receptor-expressing neurons, projecting to distinct striatal and midbrain subregions. Our experiments reveal that AAVRG injection in the nucleus accumbens (NAcc) or dorsal striatum (dSTR) of D1Cre mice labeled distinct neuronal subpopulations in medial orbitofrontal or prelimbic PFC, respectively. However, AAVRG injection in NAcc or dSTR of D2Cre mice labeled medial orbitofrontal, but not medial prelimbic PFC, respectively. Additionally, D2R+ but not D1R+ PFC neurons were labeled on injection of AAVRG in substantia nigra pars compacta (SNpc). Thus, our data are the first to highlight a unique dopamine receptor-specific topographical pattern in the PFC, which could have profound implications for corticostriatal signaling in the basal ganglia.

## Significance Statement

Cortical dopamine is critical for motivation and cognition, and its dysfunction is implicated in multiple psychiatric disorders. Cortical dopamine mediates its effects through dopamine receptors expressed on neurons that connect to multiple brain regions. Our data highlight that dopamine receptor-expressing pyramidal neurons in the cortex are more organized than previously thought. Dopamine receptor-expressing pyramidal cortical neurons are organized in distinct subcircuits that project to different target brain regions. Our findings will help us better understand the regional and global effects of cortical dopamine and its receptors, and how these discrete pathways regulate distinct dopamine-dependent functions such as reward, movement, and cognition.

## Introduction

Dopamine regulates normal processes such as motivation, reinforcement-based learning, reward and movement (Schultz, 2002; Palmiter, 2008), and its dysfunction is implicated in many psychiatric and neurologic disorders such as schizophrenia, Parkinson’s disease, obsessive compulsive disorder, attention deficit hyperactivity disorder (ADHD), and addiction (Robinson et al., 2006; Sulzer, 2011; Yager et al., 2015; Zhai et al., 2018; Abi-Dargham, 2020).

The striatum is the main input center of the basal ganglia, receiving input from cortical, thalamic, limbic and dopaminergic nuclei (Ikemoto and Bonci, 2014). Glutamatergic signals from cortical areas, and dopamine signals from midbrain dopaminergic nuclei act on spiny projection neurons (SPNs) in the striatum. The SPNs integrate both glutamate and dopamine signals and coordinate various aspects of learning and behavior (Horvitz, 2009; Shiflett and Balleine, 2011; Bamford et al., 2018).

Afferent cortical glutamatergic inputs into the striatum originate within various medial or lateral subregions of the prefrontal cortex (PFC) such as the prelimbic, infralimbic (IL), orbitofrontal, and motor cortex. The PFC plays a critical role in motivation and cognition (Balleine and O'Doherty, 2010; Smith and Graybiel, 2014). Within the medial and lateral PFC, topographically-organized regions such as the dorsally located prelimbic corticostriatal neurons, and the ventral IL or orbitofrontal PFC (OFC) neurons have dissociable effects on motivated behavior and cognitive flexibility (Killcross and Coutureau, 2003; Rudebeck and Murray, 2011; Ahmari et al., 2013; Burguière et al., 2013; Gremel and Costa, 2013; Barker et al., 2017; Hart et al., 2018). Although, these studies elegantly outline the role of sub-regions in the PFC or striatum, few studies have explored the neuronal and molecular diversity of PFC pyramidal neurons involved in regulating motivation and cognition.

Dopamine and its receptors in the PFC also regulate motivated behavior and cognitive flexibility (Goldman-Rakic, 1998; Hitchcott et al., 2007; Barker et al., 2013; Natsheh and Shiflett, 2018; Ott and Nieder, 2019). Dopamine activates D1 and D2 class of receptors in the PFC that signal through stimulatory Gαs or inhibitory Gαi proteins, respectively, and through β-arrestins as well, which modulate the activity of both pyramidal neurons and interneurons (Tseng and O'Donnell, 2004, 2007; Beaulieu et al., 2007; Santana et al., 2009; Urs et al., 2016; Ferguson and Gao, 2018; Tomasella et al., 2018; Cousineau et al., 2020). Moreover, pharmacological targeting of D1Rs or D2Rs, or genetic deletion of D2Rs in the PFC can regulate dopamine-dependent behaviors such as locomotion, cognition, and goal-directed behavior (Del Arco et al., 2007; Hitchcott et al., 2007; Barker et al., 2013; Urs et al., 2016; Natsheh and Shiflett, 2018; Tomasella et al., 2018; Khlghatyan and Beaulieu, 2020). Although PFC dopamine receptors play an important role in motivation and cognition, the topographical distribution of D1R+ or D2R+ pyramidal neurons in specific subregions of the PFC is not known. Here, we use a retrograde adeno-associated virus (AAVRG)-based approach to identify distinct topographically-organized subpopulations of D1R+ and D2R+ pyramidal neurons in the PFC based on their target projection areas. Given the role of various subregions of the PFC in motivated behavior and cognition, and the heterogeneity of these regions, the effects of dopamine and its receptors within these subregions will expand our understanding of the molecular and neuronal mechanisms regulating motivated behavior and cognition.

## Materials and Methods

### Animals

All mouse studies were performed according to NIH guidelines for animal care and use, and were approved through the University Animal Care and Use Committee. All mice were housed in a 12/12 h light/dark cycle at a maximum of five per cage, provided with food and water *ad libitum*, and tested at 8–12 weeks of age. Mice were age matched and mice of both sexes were used, and all experiments were performed in naive animals. Dopamine D1 receptor Cre (D1Cre, EY262) and Dopamine D2 receptor Cre (D2Cre, ER44) transgenic mice were obtained from MMRRC. Cre+ hemizygous transgenics were used for all experiments.

### Viral surgeries

D1Cre or D2Cre mice were stereotaxically injected unilaterally with 40- to 100-nl AAVRG, AAVRG-CAG-Flex-TdTomato (Addgene #238306) and AAVRG-hSyn-DIO-GFP (Addgene #50457). Stereotaxic coordinates are as follows: dorsal striatum (dSTR; AP +1.0, ML +1.8, DV −3.25), nucleus accumbens (NAcc) core (AP +1.0, ML +1.0, DV −4.75), dorsomedial striatum (DMS; AP +1.0, ML +1.2, DV −2750), dorsolateral striatum (DLS; AP +1.0, ML +2.2, DV −3.25); and substantia nigra pars compacta (SNpc; AP +3.5, ML +1.25, DV −4.1). Mice were allowed to recover for two weeks to allow for viral expression of GFP or TdT before imaging and counting of cells.

### Immunostaining, imaging, and quantification

40 μm thick vibratome cut sections of formalin-fixed mouse brains were processed for imaging. Sections from rostral, rostrocaudal, and caudal PFC, and striatal and midbrain sections were imaged using a Nikon AZ100 Zoom microscope, using the same exposure across genotypes for an injection pair. Captured images were used for quantifying number of fluorescent cells for each channel (GFP and TdT) in different subregions of rostral, rostrocaudal, and caudal PFC using ImageJ (NIH), and threshold was kept the same between genotypes and injection pairs. At least three sections from rostral, rostrocaudal, and caudal PFC were analyzed for each mouse, with an *n* = 5 mice per injection pair. For glutamatergic or GABAergic marker identification, we performed antigen retrieval in citrate buffer at 80°C on virally injected PFC sections, and colabeled with antibodies to GFP (Frontier Institute, catalog #AB_2571575), RFP (Rockland, catalog #600-401-379), CamKIIa (Enzo Life Sciences, catalog #ADI-KAM-CA002-D), parvalbumin (PV; Frontier Institute, catalog #AB_2571613), and GAD 65/67 (Frontier Institute, catalog #AB_2571698). Imaging for PV and GAD colabeling were done using the Nikon AZ100 zoom microscope. Imaging for CamKIIa labeling was done using a Nikon spinning disk confocal (CSU-X1, Yokogawa) with either 10× or 60× objective on an inverted microscope (Nikon Ti2-E), with a back-thinned sCMOS camera (Prime 95B, Photometrics).

### Statistical analyses

Data were analyzed by a standard two-way ANOVA test for comparison between genotypes, and injection pairs. Individual genotypes, or injection pairs were compared using a *post hoc* Tukey’s test. Data are presented as mean ± SEM, *p* < 0.05 is considered as significant.

## Results

For this study, we focused primarily on dopamine-related subcortical target regions such as the striatum and midbrain dopamine nuclei, i.e., SNpc. The striatum itself can be topographically divided along the dorsoventral or mediolateral axes, into the dorsal and ventral striatum, or DMS and DLS, respectively.

### Dorsoventral topographical distribution of D1R+ and D2R+ neurons in the PFC

**Figure 1.**
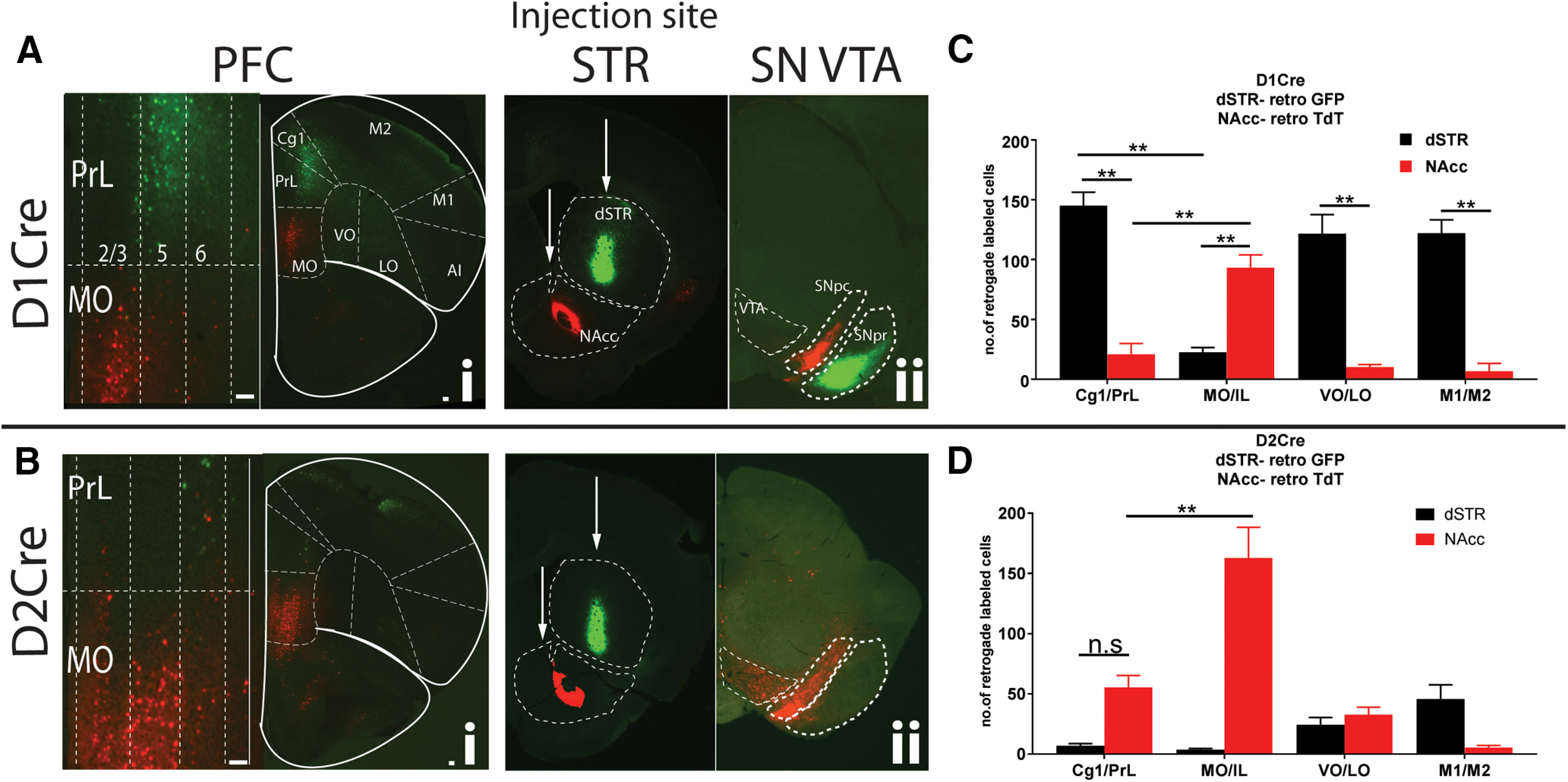
Unique topographically organized subpopulations of D1R+ and D2R+ PFC neurons project to dSTR and NAcc. PFC sections from D1Cre (***Ai***) and D2Cre (***Bi***) mice showing cell bodies retrograde labeled with both TdTomato and GFP. Left panels, Zoomed insets of medial PrL and MO showing layer-specific localization of cell bodies. PrL, prelimbic; Cg1, anterior cingulate; MO, medial orbitofrontal; VO, ventral orbitofrontal; LO, lateral orbitofrontal; AI, agranular Insular; M1, primary motor; M2, secondary motor cortex. ***Aii***, ***Bii***, Injection sites are shown in striatal (STR) sections along with midbrain SNpc/VTA (SN VTA) sections. D1Cre and D2Cre mice were injected with Cre-dependent retrograde AAV, AAVRG-CAG-Flex-TdTomato, and AAVRG-hSYN-DIO-GFP in dSTR (100 nl) and NAcc (100 nl). Representative images (*n* = 5). ***C***, ***D***, Quantification of number of cells. Data are represented as mean ± SEM; n.s., not significant; **p < 0.01, two-way ANOVA. Scale bar: 100 μm.

We first injected AAVRGs in topographically distinct target regions along the dorsoventral axis in the dSTR and the NAcc core within the ventral striatum, respectively, of either D1Cre or D2Cre mice. A total of 100 nl of AAVRG-CAG-Flex-TdTomato and AAVRG-hSyn-DIO-GFP were stereotaxically injected in the dSTR or NACC core (for coordinates, see Materials and Methods), respectively. As shown in Figure 1, we observed distinct topographically-organized patterns of GFP (dSTR) and TdT (NAcc core) positive cells in the PFC of D1Cre and D2Cre mice. For D1Cre mice, AAVRG injection in the dSTR (GFP+) and NAcc (TdT+) labeled distinct minimally overlapping subpopulations of dorsally located layer 5, and ventrally located layer 2/3 neuronal cell bodies in the PFC, respectively (Fig. 1*Ai*). The dSTR projecting neurons were primarily localized to dorsally located prelimbic/cingulate (Cg1/PrL) and motor cortex (M1/M2), and mediolaterally located ventral/lateral OFC (VO/LO), whereas the NAcc projecting neurons were primarily localized to the ventrally located medial OFC and IL (MO/IL) regions. Quantifying the number of cell bodies in these regions from rostral, rostrocaudal, and caudal PFC sections (see Materials and Methods) revealed minimal overlap (∼5%) between the PrL and MO/IL subpopulations (**p < 0.01, two-way ANOVA; Fig. 1*C*). “D1-dSTR,” Cg1/PrL: 145 ± 11.3; MO/IL: 22.5 ± 3.9; VO/LO: 121.7 ± 16; M1/M2: 122 ± 11.2 neurons, and “D1-NAcc,” Cg1/PrL: 20.8 ± 9.1; MO/IL: 93.4 ± 10.6; VO/LO: 10.1 ± 2.2; M1/M2: 6.7 ± 6.6 neurons. In contrast, for D2Cre mice, AAVRG injection in the dSTR and NAcc labeled predominantly ventrally located TdT+ layer 5 neuronal cell bodies in MO/IL, but few dorsally located GFP+ neurons in Cg1/PrL (Fig. 1*Bi*). Quantifying the number of cell bodies in these regions revealed minimal overlap of TdT+ neurons (∼15%) between the PrL and MO/IL subpopulations (**p < 0.01, two-way ANOVA; Fig. 1*D*). The NAcc projecting neurons were primarily localized to the ventrally located MO and IL (MO/IL) regions, whereas the few dSTR projecting neurons were primarily localized to M1/M2 and VO/LO regions. “D2-dSTR,” Cg1/PrL: 7 ± 1.8; MO/IL: 3.7 ± 1; VO/LO: 24.4 ± 5.9; M1/M2: 45.7 ± 11.8 neurons, and “D2-NAcc,” Cg1/PrL: 55.7 ± 9.7; MO/IL: 162.9 ± 25.5; VO/LO: 32.8 ± 6.1; M1/M2: 5.6 ± 1.7 neurons. Thus, in the medial PFC of D2Cre mice, MO/IL neurons were predominantly labeled, but not Cg1/PrL neurons.

In both D1Cre and D2Cre mice, distinct projection fibers were observed in midbrain regions, i.e., SNpc and substantia nigra pars reticulata (SNpr). In the D1Cre mice, GFP+ projection fibers (from dSTR) were observed in the SNpr, whereas, TdT+ projection fibers were observed in the SNpc (Fig. 1*Aii*). In contrast, for the D2Cre mice only TdT+ projection fibers were observed in the SNpc, and no labeling in the SNpr (Fig. 1*Bii*).

PFC projection neurons are primarily glutamatergic, but some studies have shown that a small percent of these projection neurons can be GABAergic (Lee et al., 2014; Melzer et al., 2017), and contribute to physiological outcomes. To confirm whether these retrogradely labeled neurons are glutamatergic or GABAergic, we performed colocalization studies with known glutamatergic neuron marker CamKIIa, and known GABAergic neuron markers GAD 65/67 and parvalbumin (PV). As seen in Extended Data Figure 1-1, retrogradely labeled neurons in D1 or D2Cre mice predominantly colocalize with CamKIIa but not with GAD or PV, thus confirming a glutamatergic identity of these corticostriatal projection neurons.

**Figure 2.**
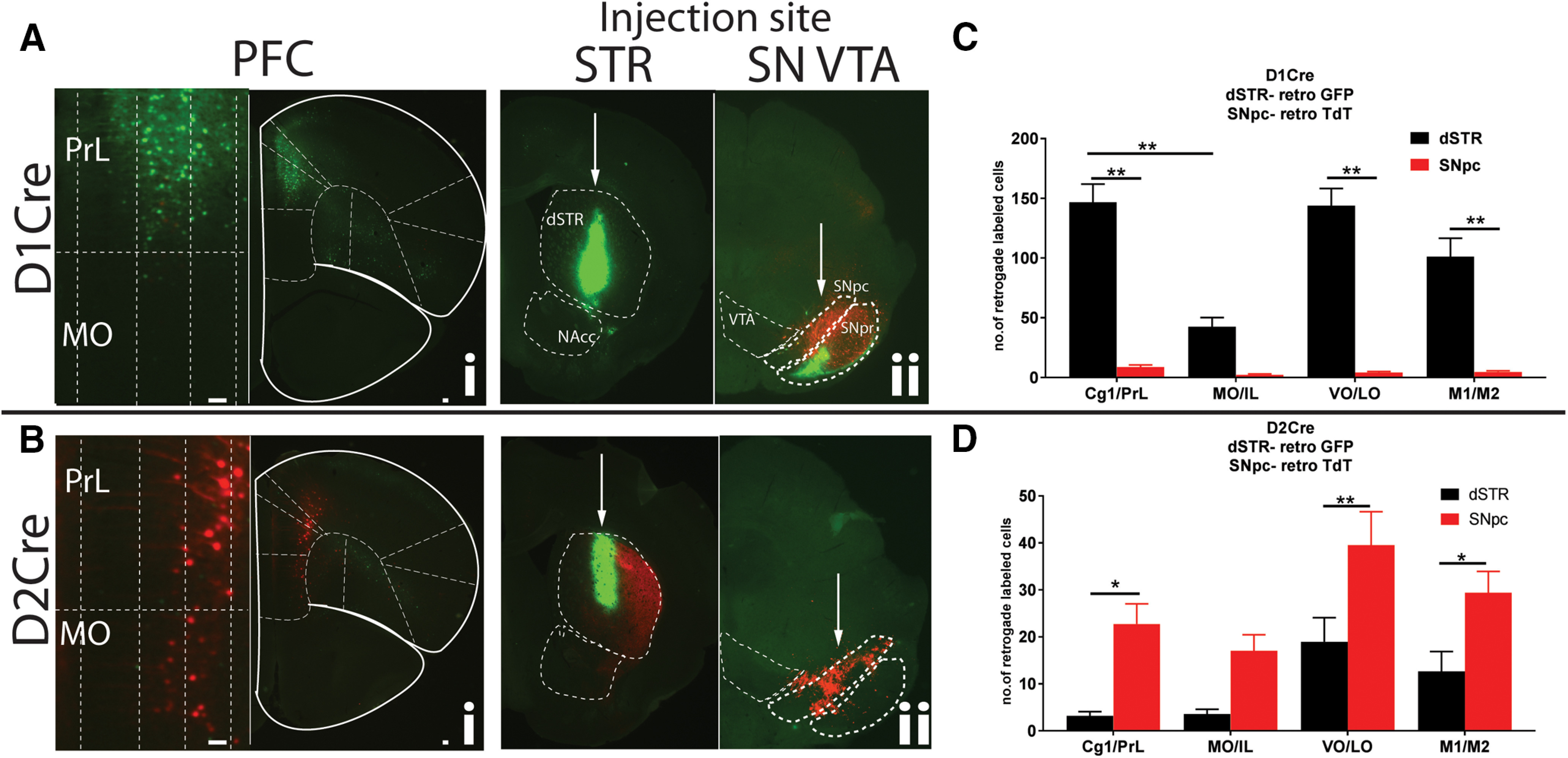
Unique topographically organized subpopulations of D1R+ and D2R+ PFC neurons project to dSTR and SNpc. PFC sections from D1Cre (***Ai***) and D2Cre (***Bi***) mice showing cell bodies retrograde labeled with either GFP or TdTomato, respectively. Left panels, Zoomed insets of medial PrL and MO showing layer-specific localization of cell bodies. PrL, prelimbic; Cg1, anterior cingulate; MO, medial orbitofrontal; VO, ventral orbitofrontal; LO, lateral orbitofrontal; M1, primary motor; M2, secondary motor cortex. ***Aii***, ***Bii***, Injection sites are shown in striatal (STR) and midbrain SNpc/VTA (SN VTA) sections. D1Cre and D2Cre mice were injected with Cre-dependent retrograde AAV, AAVRG-CAG-Flex-TdTomato, and AAVRG-hSYN-DIO-GFP in dSTR (100 nl) and SNpc (40 nl). Representative images (*n* = 5). ***C***, ***D***, Quantification of number of cells. Data are represented as mean ± SEM; *p < 0.05, **p < 0.01, two-way ANOVA. Scale bar: 100 μm.

PFC pyramidal neurons also send projections to midbrain regions, specifically to dopamine nuclei (Watabe-Uchida et al., 2012; Murugan et al., 2017). However, whether D1R+ and D2R+ PFC neurons send projections to midbrain dopamine neurons is not known. We injected AAVRGs in dSTR (GFP) as reference, and in SNpc (TdT) of D1Cre or D2Cre mice. For D1Cre mice, similar to Figure 1, injection in the dSTR labeled predominantly distinct Cg1/PrL, VO/LO and M1/M2 localized populations of PFC (Fig. 2*Ai*). However, for SNpc injections we saw minimal labeling of PFC neurons (Fig. 2*Ai*). Quantification of neurons from these regions show a similar predominant labeling of GFP+ neurons in Cg1/PrL as in Figure 1 (**p < 0.01, two-way ANOVA; Fig. 2*B*), but minimal TdT+ (SNpc) labeling. In contrast, for the D2Cre mice we observed minimal GFP+ labeling similar to Figure 1, but we observed robust TdT+ (SNpc) labeling of PFC neurons (Fig. 2*Bi*). TdT+ SNpc labeling was presumably from dopamine neurons since dense TdT+ axonal projections were seen in the dSTR (Fig. 2*Bii*). Quantification of labeled neurons from these regions revealed however, that there was no topographical pattern for the midbrain projecting D2R+ neurons (**p < 0.01, two-way ANOVA; Fig. 2*D*). “D2-SNpc,” Cg1/PrL: 22.7 ± 4.3; MO/IL: 17.0 ± 3.4 VO/LO: 39.6 ± 7.1; M1/M2: 29.4 ± 4.5 neurons. Similar patterns were observed for ventral tegmental area (VTA) injections for both D1Cre and D2Cre mice as well (Extended Data Fig. 2-1). Together, these data suggest that predominantly D2R+ and not D1R+ PFC neurons project to midbrain dopamine neurons.

10.1523/ENEURO.0194-20.2020.f1-1 Extended Data Figure 1-1 Extended Data Figure 1-1. Colabeling of D1R+ and D2R+ PFC neuron subpopulations with glutamatergic and GABAergic markers. PFC sections from D1 and D2Cre mice (*n* = 2) previously retrogradely labeled with GFP or TdT were colabeled for CamKIIa (***A***) or PV and GAD 65/67 (***B***). Download Figure 1-1, TIF file.

10.1523/ENEURO.0194-20.2020.f2-1 Extended Data Figure 2-1 Topographical distribution of VTA projecting D1R+ and D2R+ PFC neuron subpopulations. Quantification of GFP+ (VTA projecting) cells in PFC sections along the rostrocaudal axis of D1Cre and D2Cre mice. PrL, prelimbic; Cg1, cingulate; MO, medial orbitofrontal; VO, ventral orbitofrontal; LO, lateral orbitofrontal; M1, primary motor; M2, secondary motor cortex; *n* = 3 for each group. Data are represented as mean ± SEM; *p < 0.05, **p < 0.01, two-way ANOVA. Download Figure 2-1, TIF file.

**Figure 3.**
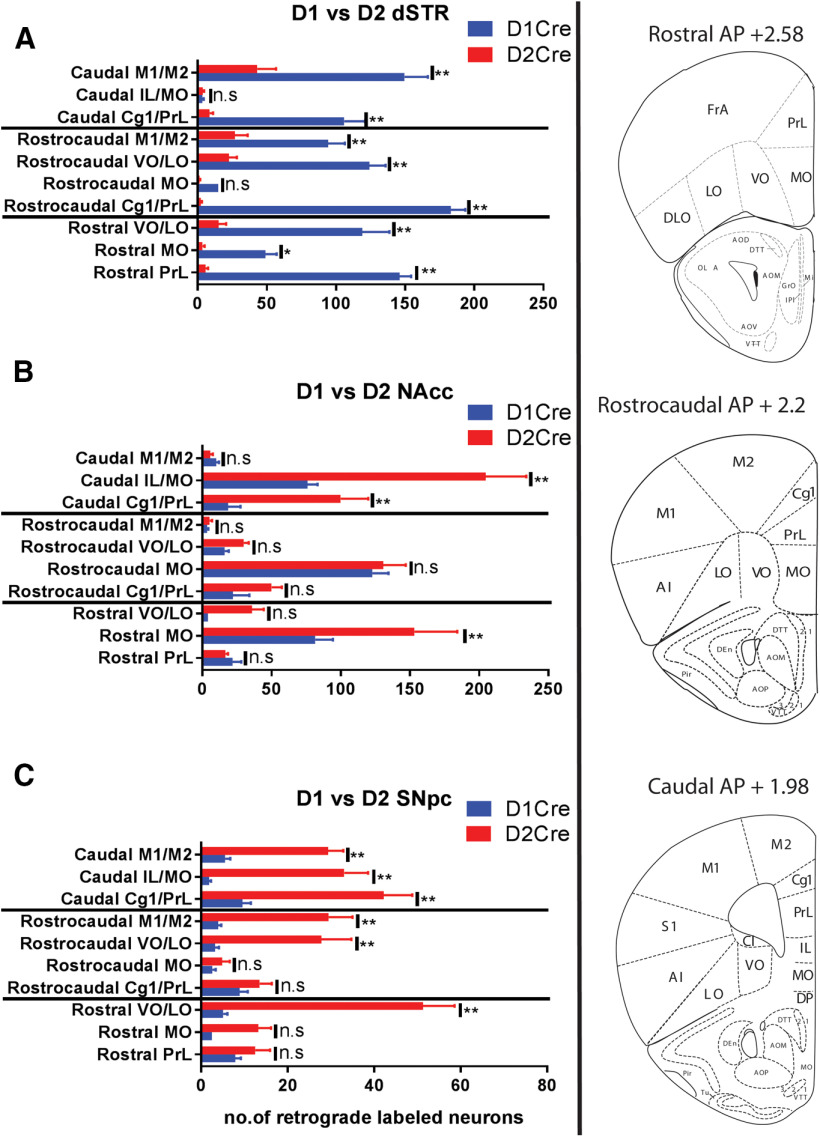
Comparison of topographical distribution of D1R+ and D2R+ PFC neurons along the rostrocaudal axis. ***A***, Quantification of GFP+ (dSTR) cells in PFC sections along the rostrocaudal axis of D1Cre and D2Cre mice. Right panel, Representative images and stereotaxic coordinates of rostral, rostrocaudal, and caudal PFC sections. ***B***, Quantification of TdT+ (NAcc core) cells in PFC sections along the rostrocaudal axis of D1Cre and D2Cre mice. ***C***, Quantification of TdT+ (SNpc) cells in PFC sections along the rostrocaudal axis of D1Cre and D2Cre mice. PrL, prelimbic; Cg1, anterior cingulate; MO, medial orbitofrontal; VO, ventral orbitofrontal; LO, lateral orbitofrontal; AI, agranular insular; M1, primary motor; M2, secondary motor cortex; *n* = 5 for each group; n.s., not significant. Data are represented as mean ± SEM; *p < 0.05, **p < 0.01, two-way ANOVA.

Figure 3 shows a more detailed comparison of PFC topographical patterns of D1Cre and D2Cre mice for each injection site, i.e., dSTR, NAcc, and SNpc, along the rostrocaudal axis, for the same mice used in Figures 1, 2. For dSTR (Fig. 3*A*), D1R+ neurons were the predominantly labeled subpopulation across the rostrocaudal axis, in all regions (Cg1/PrL, VO/LO, M1/M2) except in the MO/IL region in rostrocaudal and caudal regions. D2R+ neuron labeling ranged from 1% to 28% compared with D1R+ neurons for all regions except rostrocaudal and caudal MO/IL. For NAcc (Fig. 3*B*), both D1R+ and D2R+ neurons showed robust labeling across the rostrocaudal axis, with more D2R+ neuron labeling than D1R+ in only the rostral and caudal sections. For SNpc (Fig. 3*C*), D2R+ neurons were predominantly labeled, showing an incremental gradient along the rostrocaudal axis, whereas only 16–20% were D1R+.

### Mediolateral topographical distribution of D1R+ and D2R+ neurons in the PFC

In the previous experiments, the injection sites were along the dorsoventral axis in the dorsocentral striatum and NAcc core. However, within the dSTR, both medial and lateral subregions have specific roles in motivated behaviors. The DMS is involved in the acquisition of goal-directed actions, whereas the DLS regulates acquisition of habitual behaviors (Yin et al., 2005, 2006). Previous studies however show that mPFC pyramidal neurons primarily project to the DMS whereas more posterior lateral sensorimotor cortex neurons project to the DLS (Shiflett and Balleine, 2011; Kupferschmidt et al., 2017).

**Figure 4.**
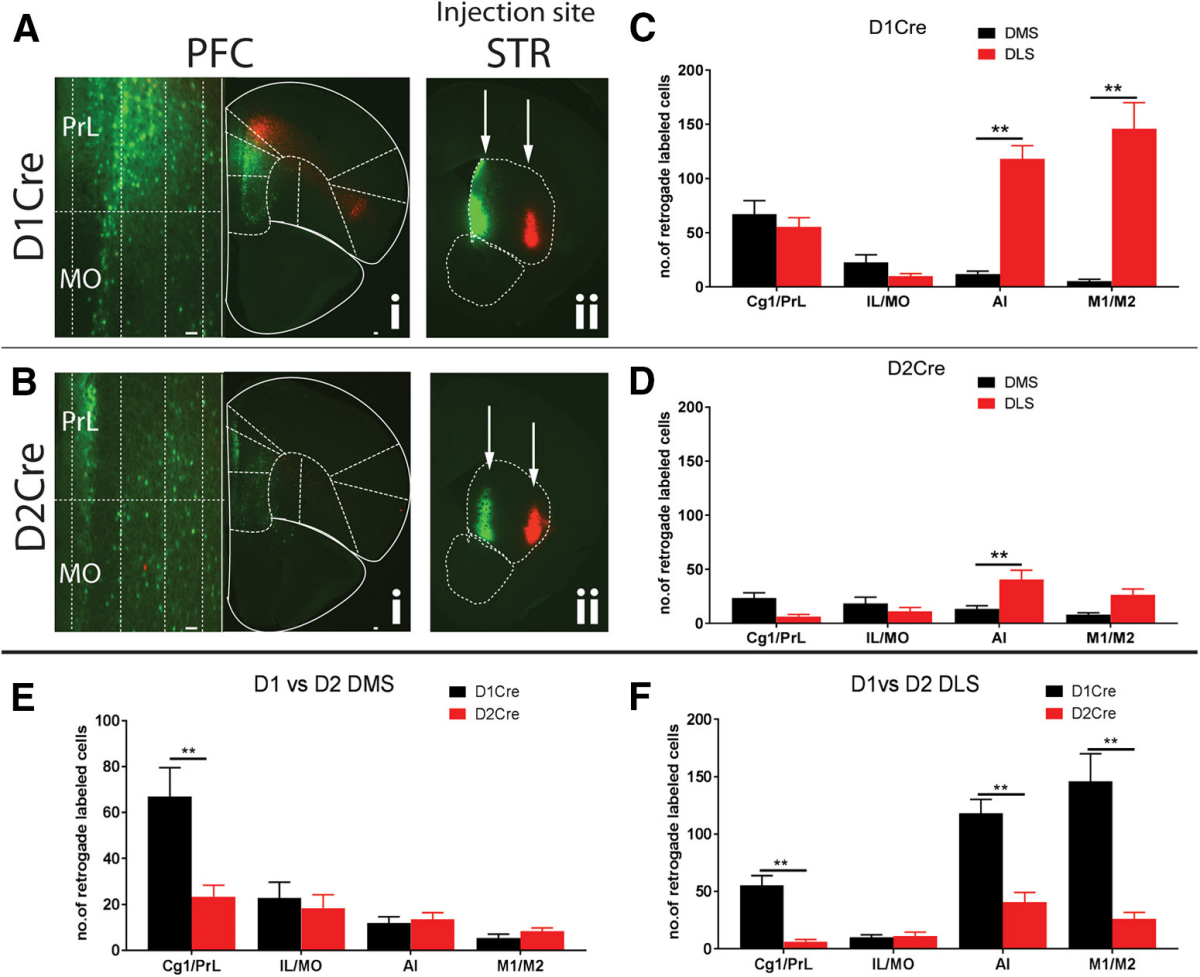
Topographical organization D1R+ and D2R+ neurons projecting to DMS and DLS. PFC sections from D1Cre (***Ai***) and D2Cre (***Bi***) mice showing cell bodies retrograde labeled with either GFP or TdTomato, respectively. Left panels, Zoomed insets of medial PrL and MO showing layer-specific localization of cell bodies. PrL, prelimbic; Cg1, cingulate; MO, medial orbitofrontal; AI, agranular insular; M1, primary motor; M2, secondary motor cortex. ***Aii***, ***Bii***, Injection sites are shown in DMS and DLS subregions in striatal sections. D1Cre and D2Cre mice were injected with Cre-dependent retrograde AAV, AAVRG-CAG-Flex-TdTomato, and AAVRG-hSYN-DIO-GFP in DMS (50 nl) and DLS (50 nl). Representative images (*n* = 5). ***C***, ***D***, Quantification of number of cells from D1Cre and D2Cre mice. ***E***, ***F***, Comparison of number of labeled cells in PFC of D1 and D2Cre mice projecting to DMS and DLS subregions. Data are represented as mean ± SEM; *p < 0.05, **p < 0.01, two-way ANOVA. Scale bar: 100 μm.

We next asked the question whether PFC D1R+ and D2R+ neurons have specific projection pattern to the DMS or DLS. Similar to Figures 1, 2, we injected 50 nl of Cre-dependent AAVRG GFP and AAVRG TdT in the DMS and DLS, respectively, of D1Cre and D2Cre mice. As seen in Figure 4, we observe distinct topographical patterns for D1R+ and D2R+ neurons projecting to DMS and DLS. For DMS injection in D1Cre mice, we observed robust GFP+ labeling predominantly in the PrL (67 ± 12.5 neurons), whereas for DLS injection we observed robust TdT+ labeling predominantly in M1/M2 (118.3 ± 12 neurons) and AI (146 ± 24.1 neurons; Fig. 4*A*,*C*). Overall, very few D2R+ neurons project to either DMS or DLS (Fig. 4*B*,*D*). Comparison of patterns of D1Cre and D2Cre mice for DMS show that only Cg1/PrL has significantly greater labeling of D1R+ neurons (**p < 0.01, two-way ANOVA; Fig. 4*E*). For DLS, however, Cg1/PrL, AI, and M1/M2 show significantly greater labeling for D1Cre compared with D2Cre mice (**p < 0.01, two-way ANOVA; Fig. 4*F*).

## Discussion

**Figure 5.**
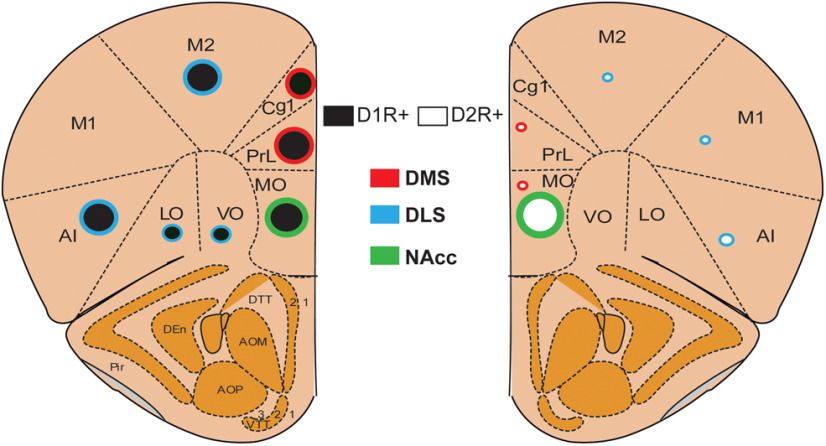
Summary of topographical organization of D1R+ and D2R+ pyramidal neurons in mouse PFC. D1R+ neurons represented by black circles and D2R+ neurons are represented by white circles. Circle borders represent corticostriatal projection targets. Size of the circle represent abundance of projections to target regions.

D1R+ and D2R+ neurons are found throughout all PFC subregions (Santana and Artigas, 2017; Anastasiades et al., 2019; Yu et al., 2019; Khlghatyan and Beaulieu, 2020), but this widespread expression pattern does not adequately explain how these neurons mediate distinct physiological and behavioral outcomes. In this study, we show a previously unappreciated distinct topographical organization of D1R+ and D2R+ neurons in the PFC of mice. As summarized in Figure 5, we observe distinct topographical organization patterns of D1R+ and D2R+ neurons along the dorsoventral and mediolateral axes, based on their projection target. Along the dorsoventral axis, PFC D1R+ neurons are topographically organized such that D1R+ neurons in prelimbic regions primarily project to the dSTR, whereas D1R+ neurons in MO and IL regions primarily project to the NAcc core. In contrast, PFC D2R+ neurons have a distinct pattern of organization, such that very few prelimbic D2R+ neurons project to dSTR, but MO and IL D2R+ neurons project to NAcc core, similar to D1R+ neurons. Along the mediolateral axis, D1R+ neurons in the medial prelimbic region primarily project to DMS, whereas D1R+ neurons in M1/M2 motor and agranular insular cortex primarily project to DLS. In contrast, very few D2R+ neurons project to either DMS or DLS. However, medial PFC D2R+ but not D1R+ neurons project to midbrain dopamine nuclei. Thus, our data provide, for the first time a detailed insight into the anatomic organization of D1R+ and D2R+ neurons in the PFC.

In this study, we use AAVRGs to identify afferent PFC inputs into various striatal and midbrain regions. One potential caveat with using AAVRGs is that we cannot control for variability of infection at the injection site, even if we inject the same volume of AAV. However, one advantage of using AAVRGs is that target neurons can be specifically labeled in a Cre-dependent manner, and therefore label cell bodies of specific populations of neurons using transgenic Cre mice. Our data are consistent with previous findings that D1R+ neurons are primarily corticostriatal, whereas D2R+ neurons are corticostriatal and also project to more caudal regions such as the thalamus (Gee et al., 2012). Other groups have also shown that PFC D1R+ and D2R+ neurons also project to other limbic areas such as the basolateral amygdala (BLA; Jenni et al., 2017). PFC neurons projecting to BLA, NAcc, or VTA are not only distinct subpopulations but also have distinct laminar distribution (Murugan et al., 2017). In this study we see distinct laminar distribution of both D1R+ and D2R+ PFC neurons. “D1-dSTR” prelimbic neurons are predominantly localized to layer 5, whereas “D1-NAcc” MO/IL neurons are predominantly localized to layer 2/3. Interestingly, “D2-NAcc” MO/IL neurons are predominantly localized to layers 2/3 and 5.

Our data also suggest that distinct predominantly D1R+ neuron subpopulations along the mediolateral axis (Cg1/PrL vs M1/M2/AI), project to the DMS and DLS, respectively. However, D2R+ PFC neurons do not project to either DMS or DLS. Thus, D1R+ PFC neurons might be directly involved in regulation of switching between goal-directed versus habitual actions (Yin et al., 2005, 2006). Interestingly, a similar topographical pattern is maintained within the SNpc, where medial and lateral dopamine neurons project to DMS and DLS, respectively (Lerner et al., 2015).

The dSTR-NAcc injections (Fig. 1) reveal distinct projection fiber patterns in the SNpr and SNpc. For injections in dSTR, we observed projection fibers in the SNpr only in D1Cre and not D2Cre mice, consistent with AAVRG-GFP labeling striatal D1R+ direct pathway SPNs. However, for NAcc core injections, we observe projection fibers in the medial SNpc and not the SNpr in both D1Cre and D2Cre mice. Our findings are consistent with previous observations that D1R+ and D2R+ NAcc core SPNs do not follow the traditional direct/indirect dichotomy like dSTR SPNs, and instead send projections to ventral pallidum (VP) or midbrain (Sesia et al., 2014; Kupchik et al., 2015; Pardo-Garcia et al., 2019). Moreover, these studies suggest that both D1R+ and D2R+ NAcc core SPNs project to VP, but only D1R+ NAcc core SPNs project to the midbrain. Although we observe projection fibers in the SNpc of D2R+ mice, these are likely direct projections from the MO/IL PFC (Fig. 2*B*), and not from NAcc core SPNs. Thus, D1R+ pyramidal neurons in the MO/IL projecting to NAcc core, can not only release glutamate and regulate excitability of GABAergic SPNs, but also modulate dopamine release in the DMS by indirectly acting on medial SNpc dopamine neurons. In contrast, D2R+ pyramidal neuron in the MO/IL project directly to both NAcc core and SNpc. One possible caveat of this interpretation is that by using AAVRGs we are unable to establish whether these fibers in the SNpc are afferents on GABAergic or dopaminergic neurons. A more sophisticated approach with rabies virus retrograde labeling, with Cre-dependent labeling of target neurons is required to confirm our interpretation.

The dSTR is topographically divided into the DMS and DLS, which have been implicated in action-outcome learning and stimulus-response learning, respectively, whereas the NAcc has been implicated in reward perception (Shiflett and Balleine, 2011). D1R+ and D2R+ neurons in topographically organized regions in the PFC can thus have various effects on physiology and behavior depending on their striatal or midbrain projection target, and modulation by cortical dopamine.

Illuminating this unique pattern of organization of D1R+ and D2R+ neurons in the PFC will help us better understand the regional and global effects of cortical dopamine and dopamine receptors in the regulation of motivation and cognition.

## Synthesis

Reviewing Editor: Kirill Martemyanov, The Scripps Research Institute Decisions are customarily a result of the Reviewing Editor and the peer reviewers coming together and discussing their recommendations until a consensus is reached. When revisions are invited, a fact-based synthesis statement explaining their decision and outlining what is needed to prepare a revision will be listed below. The following reviewer(s) agreed to reveal their identity: Emmanuel Valjent, Yevgenia Kozorovitskiy.

Synthesis.

Upon discussion the reviewers agreed that the study provides important insights into topographical organization of the striatal projections. The reviewers noted careful nature of the study and its systematic approach filling significant area of need in the field for its fundamental insights into basal ganglia anatomy.

There was also a consensus that the study is technically sound and the data are convincing. Both reviewers made a number of comments, addressing which was felt to be important before the study could be published. Please refer to specific comments below.

Reviewer #1

The present study aims to precise the topographic organization of D1R and D2R PFC neurons projections. To perform this mapping the authors used advanced cell-type specific tracing methods allowing to establish a comprehensive map of the connectivity of D1R and D2R PFC neurons projections. The results are clear and the data are convincing.

Few points listed below need to be addressed:

Major comment:

- An important issue here is to precise the identity of D1R and D2R PFC neurons. Double immunofluorescence using CaMKII should be performed to determine the proportion of those neurons that are glutamatergic. In addition, the percentage of GFP or tdTomato-positive co-expressing PV and/or SOM should be assessed since recent studies support the exitence of long-range GABAgergic PFC neurons. These new results will strength the characterization and should be discussed in the revised version.

- Figure 1: please illustrate GFP positive cells detected in M1/M2 and VO/LO for the D1-Cre mice.

- Figure 5 is useful but should be completed by adding the location of D1R and D2R PFC neurons projecting to the SNc and SNr.

- The authors should maybe consider to change the title because the characterization goes beyond the PFC. The study also provides important insights regarding the M1/M2 cortex and Agranular insular cortex.

- Recent papers related to D2R cortical neurons should be quoted and discussed (Khlghatyan et al., 2018 Cerebral Cortex; Cousineau et al., 2020, eNeuro).

- Discussion. The sentence related to the work of Kupchik is confusing. ID1R an D2R SPNs of the Acb Core project both to the VP but only D1R project to SNr. This should be clarified.

Minor comment:

- In the third paragaph of the introduction, the authors should mention that D2Rs also signal through β-arrestin.

- In the method section, the authors flipped the reference of the mouse lines. D2R-Cre is the ER44 and D1R-Cre is the EY262.

Reviewer #2

This study relies on Cre-dependent retrograde labeling to compare projections from D1 and D2 receptor-expressing neurons from the mPFC. The data demonstrate that the dorsal striatum (dSTR) and the nucleus accumbens (NAcc) receive differential cortical inputs from the medial prefrontal cortex (mPFC). Overall, the authors provide some fundamental anatomical evidence showing that cortical D1 and D2 populations target different subregions in striatum, and dSTR and NAcc receive inputs from preferentially from specific subregions and DA-sensing neurons of mPFC. There are a few moderate and minor comments for this generally useful for the field data set.

Major concern:

1) The authors suggest that there is a dorsoventral “topographical” distribution of D1+ and D2+ in the mPFC. In their data, the D1+ inputs to striatum are from both PrL and MO, while D2+ inputs are mainly from MO, targeting ventral striatum. There are two interpretations: a) The inputs to a subregion in striatum depend on both receptor type (D1 vs D2), and subregion in mPFC (PrL vs MO). b) The difference shown in data is observed simply because PrL projects to dSTR and MO projects to NAc, but the D1+ population distributes in both regions, while the D2+ population resides preferentially in the MO. To further justify this “topographical” specificity, the authors should also evaluate the distribution of D1 and D2 neurons in the mPFC in the two Cre lines, or find results from existing literature/data sets.

Other concerns:

1) In Figure 1aii, the authors should clarify that the SNr labeling is likely from the SPNs.

2) In Figure 2, show the VTA data.

3) Add scale bars on all images.

4) Clarify whether male, female or mice of both sexes were used.

5) Add virus addgene catalog numbers to methods section.
