# Supplementary figures and images for: Retrograde Labeling Illuminates Distinct Topographical Organization of D1 and D2 Receptor-Positive Pyramidal Neurons in the Prefrontal Cortex of Mice
Source: eNeuro. 2020 Oct 22;7(5):ENEURO.0194-20.2020. doi: 10.1523/ENEURO.0194-20.2020 (PMC7665905; doi:10.1523/ENEURO.0194-20.2020)

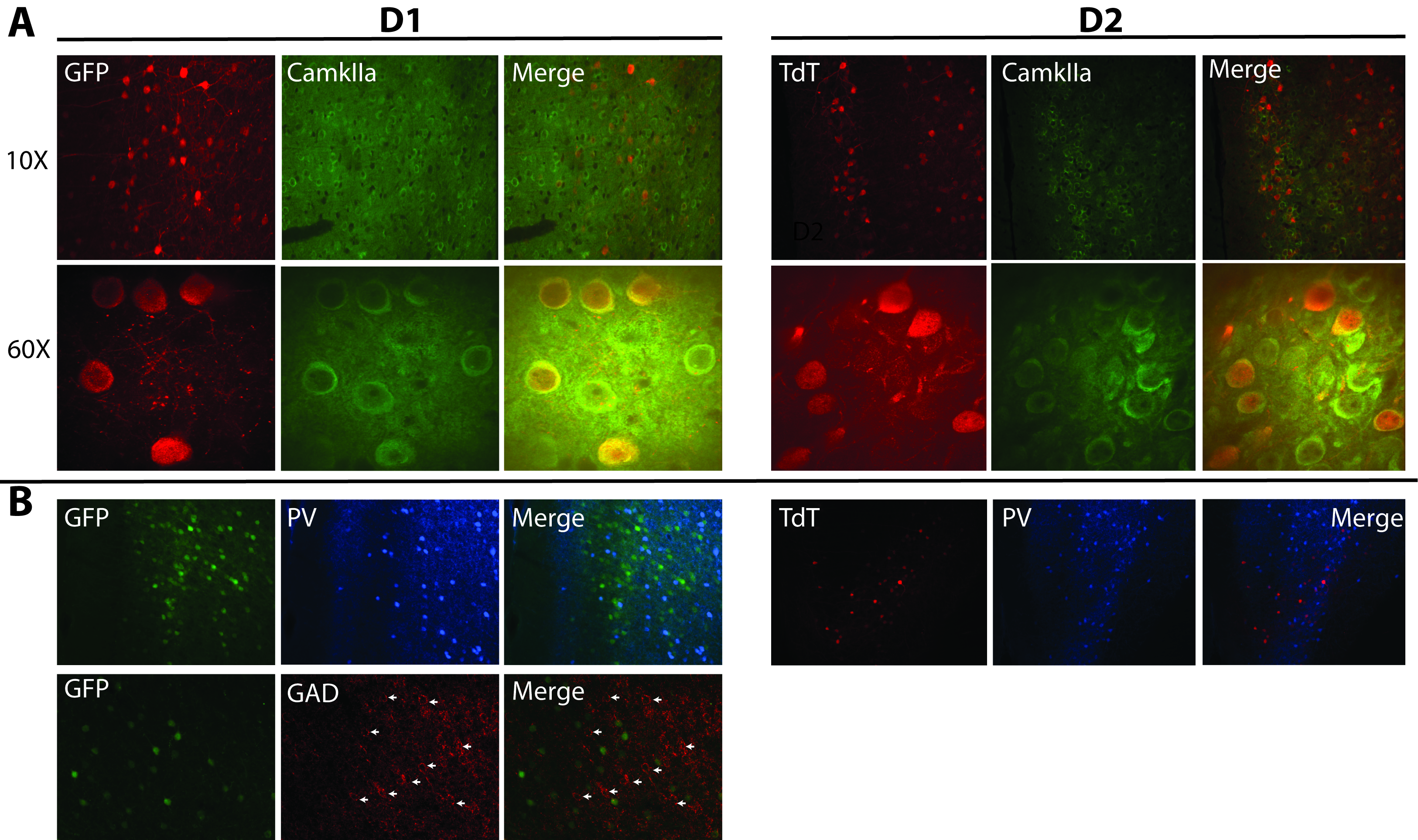

Supplement: Extended Data Figure 1-1 — Colabeling of D1R+ and D2R+ PFC neuron subpopulations with glutamatergic and GABAergic markers. PFC sections from D1 and D2Cre mice (n = 2) previously retrogradely labeled with GFP or TdT were colabeled for CamKIIa (A) or PV and GAD 65/67 (B). Download Figure 1-1, TIF file. [file enu-eN-NWR-0194-20-s02.tif]

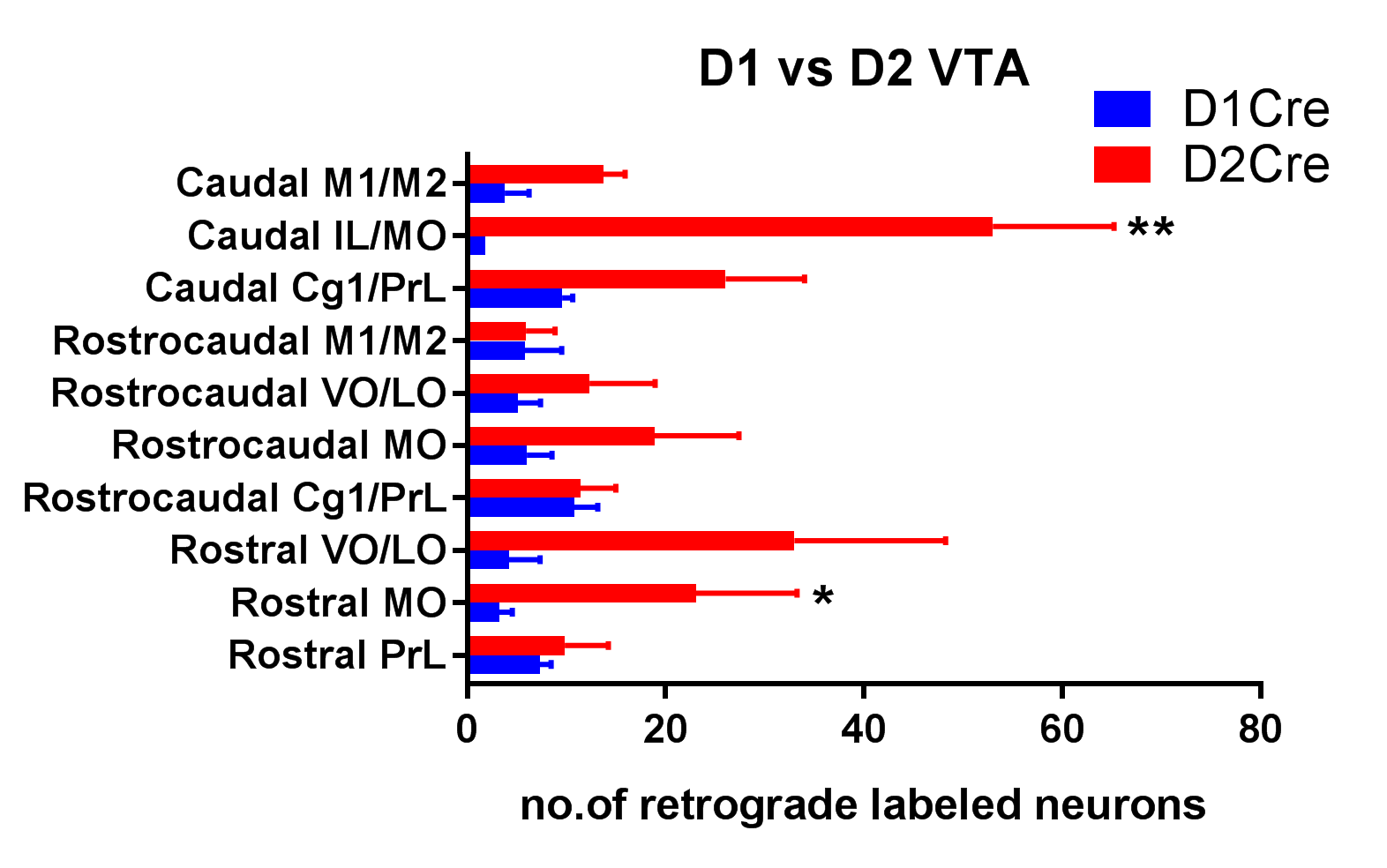

Supplement: Extended Data Figure 2-1 — Topographical distribution of VTA projecting D1R+ and D2R+ PFC neuron subpopulations. Quantification of GFP+ (VTA projecting) cells in PFC sections along the rostrocaudal axis of D1Cre and D2Cre mice. PrL, prelimbic; Cg1, cingulate; MO, medial orbitofrontal; VO, ventral orbitofrontal; LO, lateral orbitofrontal; M1, primary motor; M2, secondary motor cortex; n = 3 for each group. Data are represented as mean ± SEM; *p < 0.05, **p < 0.01, two-way ANOVA. Download Figure 2-1, TIF file. [file enu-eN-NWR-0194-20-s03.tif]
